# A genome-wide landscape of mRNAs, lncRNAs, circRNAs and miRNAs during intramuscular adipogenesis in cattle

**DOI:** 10.1186/s12864-022-08911-z

**Published:** 2022-10-06

**Authors:** Xinran Yang, Xinhao Ma, Chugang Mei, Linsen Zan

**Affiliations:** 1 College of Animal Science and Technology, Northwest A&F University, Yangling, 712100 Shaanxi China; 2 College of Grassland Agriculture, Northwest A&F University, Yangling, 712100 Shaanxi China; 3 National Beef Cattle Improvement Center, Northwest A&F University, Yangling, 712100 Shaanxi China

**Keywords:** Cattle, IMF, Preadipocyte differentiation, CircRNA, LncRNA, miRNA, RNA-seq, WGCNA, CeRNA

## Abstract

**Background:**

Intramuscular preadipocyte differentiation plays a critical role in bovine intramuscular fat (IMF) deposition. However, the roles of different RNAs, including mRNAs, circRNAs, lncRNAs and miRNAs, in regulating the adipogenic differentiation of intramuscular preadipocytes remain largely unclear.

**Results:**

In the present study, a whole transcriptome sequencing and analysis, including the analysis of mRNAs, circRNAs, lncRNAs and miRNAs, during different differentiation stages (0, 3, 6, and 9 d) of intramuscular preadipocytes from Qinchuan cattle was performed. All samples were prepared with 3 biological replicates. Here, a total of 27,153 mRNAs, 14,070 circRNAs, 7035 lncRNAs, and 427 miRNAs were annotated. Among them, we identified 4848 differentially expressed mRNAs (DEMs), 181 DE circRNAs (DECs), 501 DE lncRNAs (DELs) and 77 DE miRNAs (DEmiRs) between 0 d and other differentiation days (3, 6, and 9 d). GO and KEGG functional enrichment analyses showed that these differentially expressed genes were mainly enriched in cell differentiation, fat metabolism and adipogenesis-related pathways. Furthermore, weighted gene coexpression network analysis (WGCNA) and co-expression network analysis screened out multiple important mRNAs, circRNAs and lncRNAs related to intramuscular adipogenesis. Based on the competing endogenous RNA (ceRNA) regulatory mechanism, we finally identified 24 potential ceRNA networks and 31 potential key genes, including *FOXO1*/miR-330/circRNA2018/MSTRG.20301, *GPAM*/miR-27b/ciRNA489 and *SESN3*/miR-433/circRNA2627MSTRG.20342.

**Conclusions:**

This study provides new insights into the differential expression patterns of different transcript types (i.e., mRNAs, circRNAs, lncRNAs and miRNAs) in intramuscular preadipocyte differentiation. Our findings provide data support for studying the molecular mechanism of key mRNAs and noncoding RNAs in IMF deposition, and provide new candidate markers for the molecular breeding of beef cattle.

**Supplementary Information:**

The online version contains supplementary material available at 10.1186/s12864-022-08911-z.

## Background

Intramuscular fat (IMF), as an important component of beef marbling, directly determines the quality of beef [1, 2]. It is closely related to the tenderness, flavour and juiciness of beef [3]. The monounsaturated fatty acids contained in IMF are also more beneficial to cardiovascular health and reduce the risk of fat metabolism-related diseases [4]. IMF deposition is influenced by a variety of factors, including genetics, nutrition and management [5]. Unlike other adipose tissues, IMF deposition in animals occurs mainly from the fetal stage to weaning age. The differentiation of intramuscular preadipocytes mainly occurs in the fetal stage, while the lipid accumulation of intramuscular adipocytes mainly occurs from birth to the weaning age [6–8]. After weaning, the content of IMF is usually increased by means of enhanced nutrition and management, but it also results in the accumulation of visceral and subcutaneous fat, which can be detrimental to the health of the animal. Therefore, it is essential to study the molecular mechanism of intramuscular preadipocyte differentiation. The growth and development of IMF in beef cattle are likewise subject to complex regulation by a variety of genes. For example, the adipokine chemerin promoted the deposition of IMF in cattle by upregulating the expression of *PPARG*, *C/EBPA* and *FABP4* [9]. Single nucleotide polymorphisms in the *CDC10* gene affected the content of bovine IMF [10]. Although these studies on a single regulatory factor can provide some reference and insight, it is more important to systematically reveal the molecular regulatory network of IMF deposition in beef cattle.

Noncoding RNAs are a class of RNAs that do not have or have only weak coding ability. There is growing evidence that noncoding RNAs have regulatory abilities that are not weaker than those of coding RNAs. Three categories of important noncoding RNAs are microRNAs (miRNAs), circular RNAs (circRNAs), and long noncoding RNAs (lncRNAs), which play important roles in a variety of physiological processes, including the regulation of bovine adipose tissue growth and development through chromatin modification, transcriptional activation, and transcriptional/translational interference [11–13]. miRNAs are strongly and functionally conserved among species and were the first noncoding RNAs identified and characterized [14]. Our previous studies have shown that bta-miR-150 regulates the mTOR pathway by targeting the 3’UTR of *AKT1*, thereby affecting the proliferation and differentiation of bovine preadipocytes [15]. bta-miR-376a inhibited bovine adipocyte differentiation through the *KLF15/PPARG* axis [16]. Studies on circRNAs have mainly focused on regulatory effects through competitive endogenous RNA (ceRNA) networks. *circFUT10* has been reported to affect *PPARGC1B* expression by adsorbing let-7 and thereby inhibiting bovine preadipocyte differentiation [17]. *circINSR* indirectly affected *CCND1* and *Bcl-2* expression by binding miR-15/16, thereby regulating bovine fat development [18]. The effects of lncRNAs on bovine fat development are more diverse. For example, different spliceosomes of *lncFAM200B* differentially affected preadipocyte proliferation [19]. In addition, a newly identified *lncRNA BADLNCR1* was able to inhibit bovine adipogenesis by directly suppressing *GLRX5* expression [20].

Whole transcriptome sequencing is the simultaneous sequencing of mRNA, lncRNA, circRNA and miRNA in a sample. The results can truly reflect the actual biological processes and allow direct analysis of the interaction effects between key regulatory elements. Compared with previous sequencing analyses of one type of RNA alone, whole transcriptome sequencing eliminates the batch effect well, and its analysis results have higher accuracy. Whole transcriptome sequencing in porcine subcutaneous adipogenesis and longissimus dorsi muscle of different breeds screened a series of mRNAs/lncRNAs/circRNAs with potential regulatory functions on fat and muscle growth and development, respectively [21, 22]. However, the application of whole transcriptome sequencing technology in the field of bovine IMF deposition is still lacking, and the molecular network regulating bovine IMF deposition is still unclear. In this study, intramuscular preadipocytes isolated from the longissimus dorsi muscle of Qinchuan cattle were used as the research objects. Whole transcriptome sequencing (including circRNA-seq and miRNA-seq) was performed on intramuscular preadipocytes at Days 0, 3, 6 and 9 of adipogenic differentiation. Based on the analysis of differentially expressed genes, the differentially expressed circRNAs, lncRNAs and miRNAs were further screened through a series of bioinformatics analyses. Then, important circRNAs, lncRNAs and miRNAs with potential regulatory effects on bovine IMF deposition were finally identified by predicting the interaction regulatory networks among them. Our results contribute to an improved understanding of the molecular mechanisms of bovine IMF deposition.

## Results

### Adipogenic differentiation of intramuscular preadipocytes

**Fig. 1:**
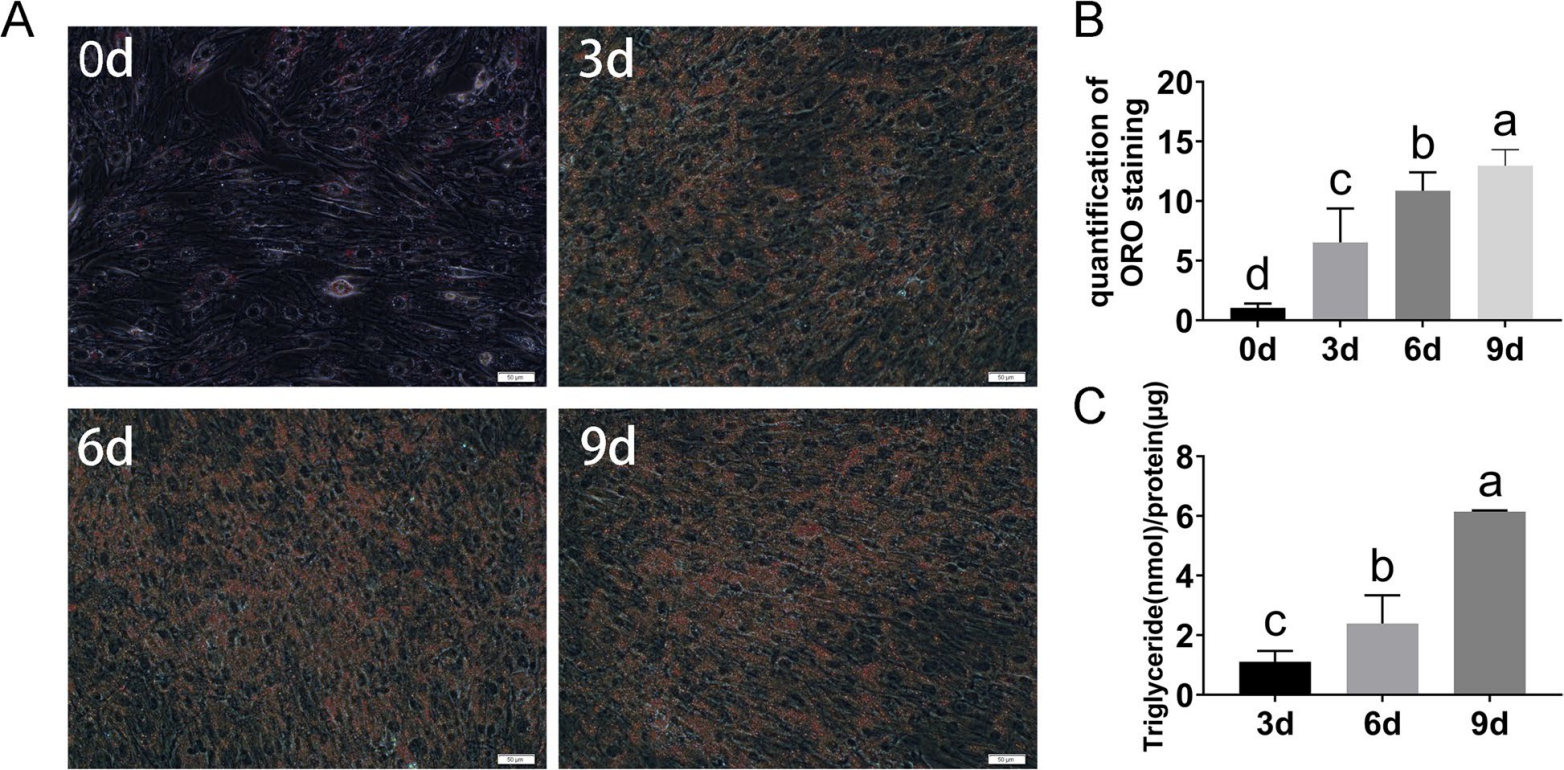
Adipogenic differentiation of bovine intramuscular preadipocytes. **A**, **B** Oil Red O staining at the different differentiation stages (**A**) and quantitative results (**B**). **C** Triglyceride content of bovine preadipocytes on days 3, 6, and 9 of differentiation. *n* = 3. Different lowercase letters indicate *P* < 0.05 and the same letters indicate *P* > 0.05

Lipid droplets in intramuscular preadipocytes gradually increased with the induction of adipogenic differentiation (Fig. 1A). Meanwhile, the results of Oil Red O quantification and triglyceride assays also showed a gradual increase in lipid droplets on Days 0, 3, 6, and 9 of adipogenic differentiation (Fig. 1B, C). These results indicated that the bovine intramuscular preadipocytes isolated in the present study exhibited excellent adipogenic differentiation ability and could be used for subsequent studies.

### Expression patterns of mRNA/circRNA/lncRNA during intramuscular preadipocyte differentiation

**Fig. 2:**
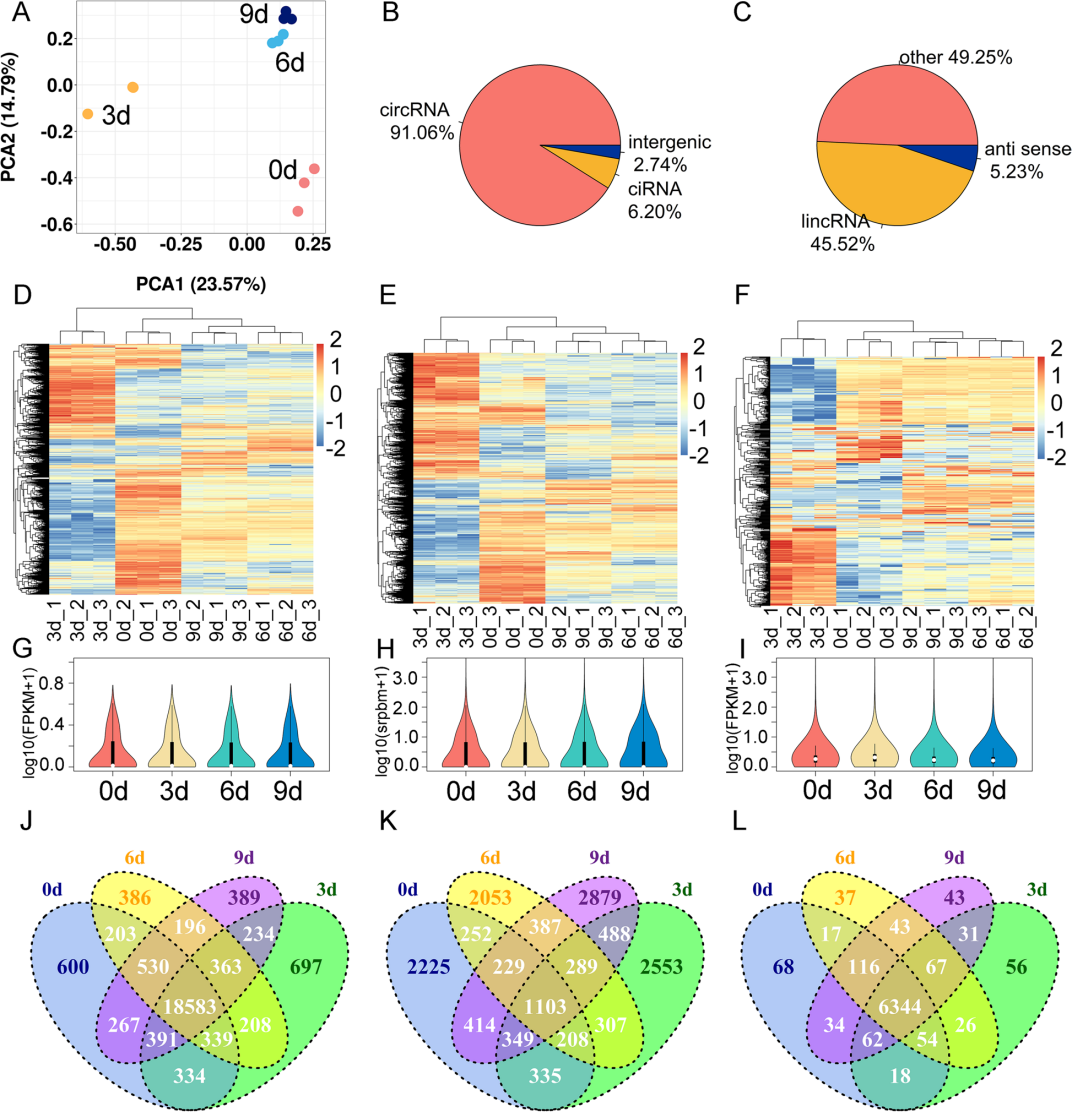
Expression patterns of mRNAs, circRNAs and lncRNAs during the adipogenic differentiation of bovine intramuscular preadipocytes. **A** PCA of four groups of samples. **B** The type and proportion of circRNAs. **C** The type and proportion of lncRNAs. **D-F** Clustered expression heatmaps of all mRNAs (**D**), circRNAs (**E**) and lncRNAs (**F**). **G-I** Violin plot of gene expression levels all mRNAs (**G**), circRNAs (**H**) and lncRNAs (**I**). **J**-**L** Venn diagram showing the specifically expressed and commonly expressed mRNAs (**J**), circRNAs (**K**), and lncRNAs (**L**) in four adipogenic differentiation stages (0d, 3d, 6d and 9d)

Total RNA from Days 0, 3, 6, and 9 of intramuscular preadipocyte differentiation were extracted for RNA-seq. Principal component analysis (PCA) found that the intragroup repeatability of the four groups of samples (0 d, 3 d, 6 d and 9d) was high, and the differences between the groups were obvious (Fig. 2A). Among them, the correlation between 6 d and 9 d was higher (Fig. 2A). RNA-seq generated 66,224,070 - 92,352,434 valid reads in all samples, of which more than 96% were mapped to the reference genome of *Bos taurus* (Additional file 1). They all had a Q30 of > 98% (Additional file 1). A total of 27,153 mRNAs, 14,070 circRNAs, and 7035 lncRNAs were annotated from four stages (0, 3, 6, and 9 d) of adipogenic differentiation (Additional file 2). Meanwhile, the identified lncRNAs contained 45.52% long intergenic noncoding RNA (lincRNAs), and 91.06% of typical circRNAs were identified (Fig. 2B, C). As shown in Fig. 2D and E, the clustered expression heatmaps of mRNAs (Fig. 2D), circRNAs (Fig. 2E) and lncRNAs (Fig. 2F) showed differences between groups and biological replicates within groups. The distribution of mRNA/circRNA/lncRNA expression was measured and displayed in a violin plot, respectively (Fig. 2G-I). In addition, the mRNAs, lncRNAs, and circRNAs annotated at each differentiation time point (0, 3, 6 and 9 d) were overlapped. We found 600, 697, 386, 389 specifically expressed mRNAs and screened 18,583 common genes (Fig. 2J). However, there were only 1103 common circRNAs and 2225, 2553, 2053, and 2879 specifically expressed circRNAs, respectively (Fig. 2K). The numbers of specifically expressed lncRNAs were even lower, only 68, 56, 37, and 43, respectively, but there were 6344 common lncRNAs (Fig. 2L).

### Differential expression analysis of mRNA/circRNA/lncRNA

**Fig. 3:**
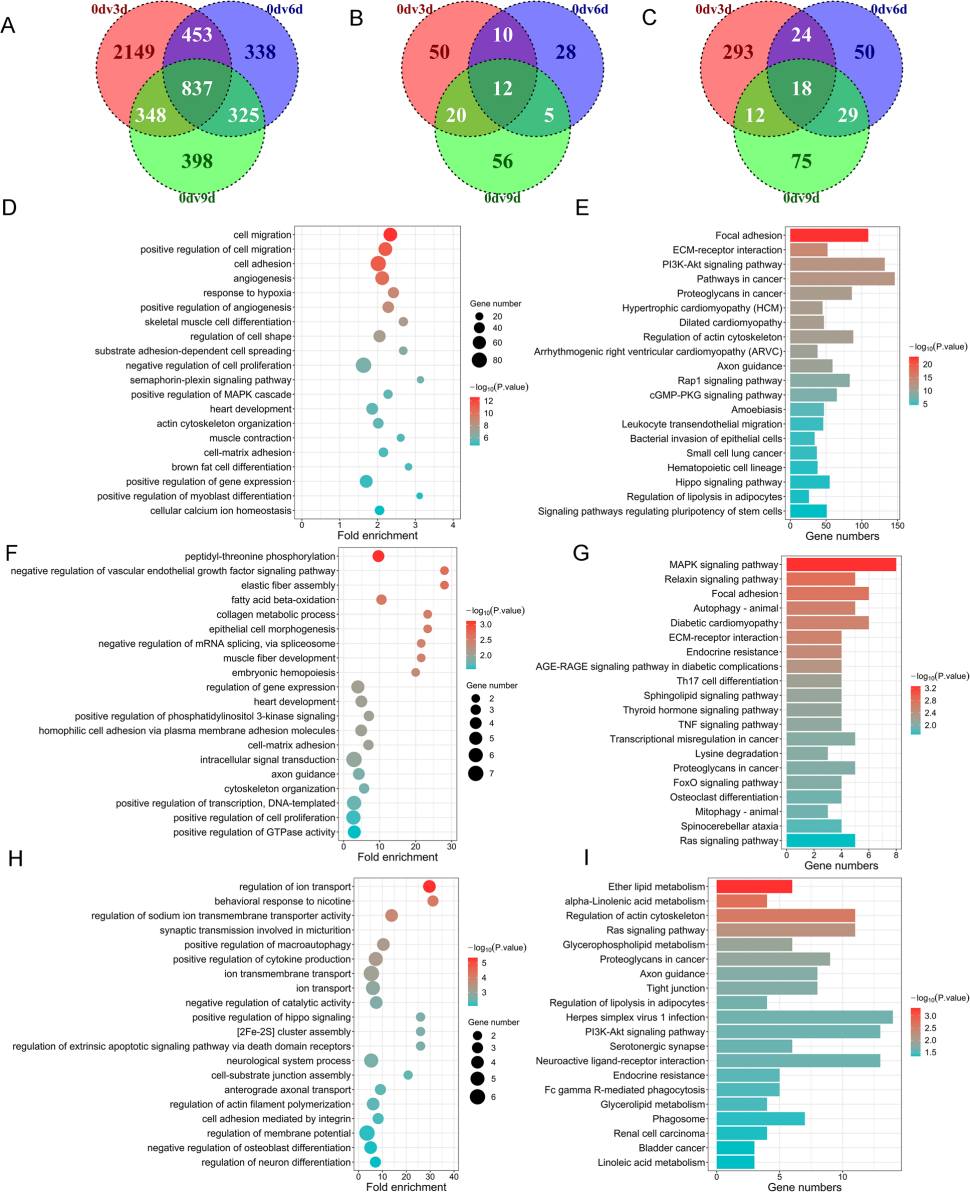
Differential expression analysis of mRNAs, circRNAs and lncRNAs. **A**-**C** Expression analysis of common DE genes during the intramuscular preadipocyte differentiation. The number of common DE mRNAs (**A**), DE circRNAs (**B**) and DE lncRNAs (**C**). **D**, **E** GO (**D**) and KEGG (**E**) enricment analysis of common DE mRNAs. **F**, **G**) GO (**F**) and KEGG (**G**) enricment analysis of common DE circRNAs. **H**, **I** GO (**H**) and KEGG (**I**) enricment analysis of common DE lncRNA

To discover the differentially expressed genes (DEGs) during adipogenic differentiation of intramuscular preadipocytes, three comparison groups (3 d vs. 0 d, 6 d vs. 0 d, and 9 d vs. 0 d) were used. Among these comparison groups, DEGs were characterized as 4848 differentially expressed mRNAs (DEMs) (Fig. 3A and Additional file 3), 181 differentially expressed circRNAs (DECs) (Fig. 3B and Additional file 4) and 501 differentially expressed lncRNAs (DELs) (Fig. 3C and Additional file 5). Subsequently, we performed functional enrichment analysis of DEMs, host genes of DECs and cis-regulated genes of DELs to further predict the potential functions of these DEGs in intramuscular adipogenesis. The results showed that the GO terms and KEGG signaling pathways of DEMs were mainly enriched in negative regulation of cell proliferation, positive regulation of the MAPK cascade, brown fat cell differentiation, the PI3K-Akt signaling pathway and Regulation of lipolysis in adipocytes (Fig. 3D, E and Additional file 6). DECs might be mainly involved in the positive regulation of cell proliferation, fatty acid beta-oxidation, the MAPK signaling pathway, the Thyroid hormone signaling pathway and the FoxO signaling pathway (Fig. 3F, G and Additional file 6). The results of functional enrichment analysis of DELs indicated that DELs might be involved in fatty acid beta-oxidation, the MAPK signaling pathway, Focal adhesion and the FoxO signaling pathway (Fig. 3H, I and Additional file 6).

### Weighted gene Coexpression network analysis (WGCNA)

**Fig. 4:**
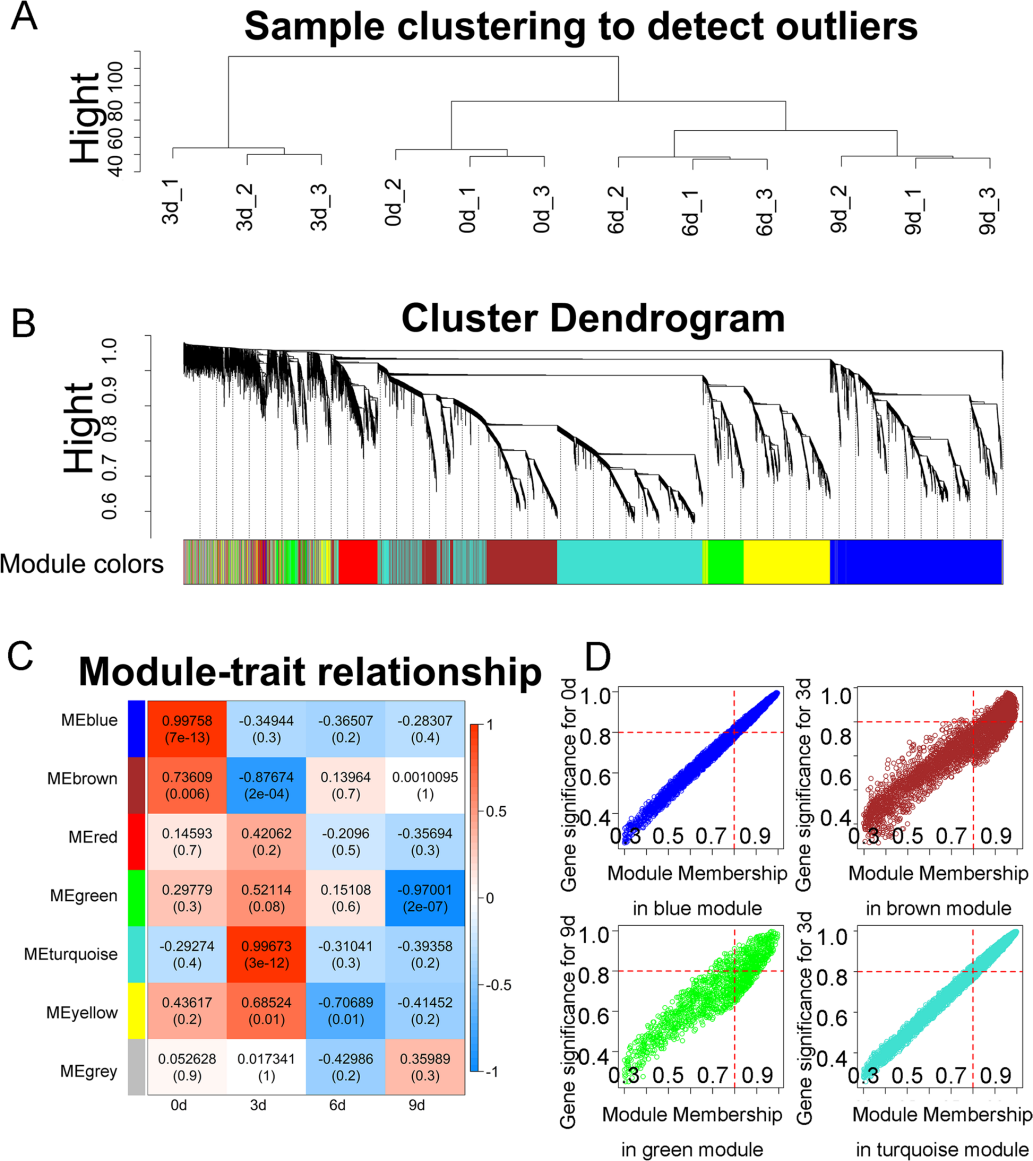
WGCNA of mRNAs. **A** Clustering information of samples. **B** The dendrogram of gene clustering was generated utilizing hierarchic clustering of adjacency-based dissimilarity. Co-expression modules detected by the dynamic hybrid-cutting approach are represented by the color blocks below the tree graph. **C** Heatmap of the correlation between module and four adipogenic differentiation stages. **D** Module Membership in blue, brown, turquoise and green

A large number of common mRNAs and DEMs were identified during the adipogenic differentiation of intramuscular preadipocytes (Fig. 2 J and Fig. 3 A), therefore, we performed WGCNA to screen the key mRNAs among them. First, with the total mRNA expression matrix as input, after cluster analysis confirmed that there were no outlier samples, a soft threshold of 4 was chosen, and the connectivity was calculated (Fig. 4A). Then, mRNAs were divided into different modules based on different expression patterns (Fig. 4B). The correlations of these modules with other modules and different differentiation stages were estimated (Fig. 4C and Additional file 7). In total, six modules were generated: turquoise, blue, brown, yellow, green and red, containing 5093, 4053, 3186, 2623, 1490 and 1221 coding genes, respectively. We selected the modules with strong correlations with different differentiation stages for further study, which included the blue, brown, turquoise and green modules. Finally, the hub genes in the blue, brown, turquoise and green modules were filtered out by calculating and restricting both gene significance (GS) and module membership (MM) values greater than 0.8 (Fig. 4D and Additional file 7). Finally, these hub genes that were differentially expressed during adipogenic differentiation and were related to lipid metabolism and cell differentiation were selected for further investigation (Additional file 7).

### Construction of the circRNA/lncRNA–mRNA coexpression network

**Fig. 5:**
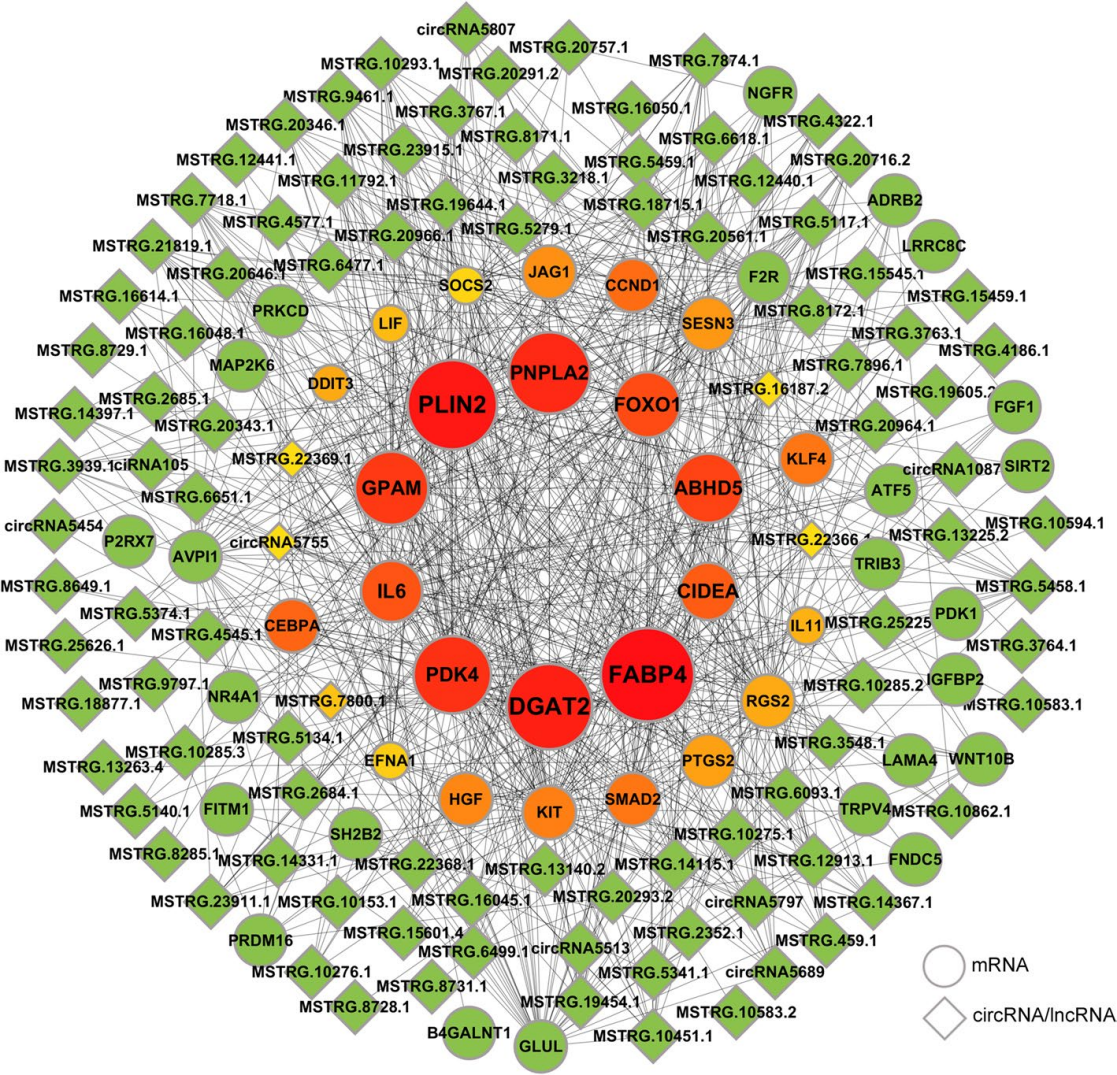
circRNA/lncRNA-mRNA coexpression network. Coexpressed network of DE circRNA/lncRNAs and their targeted DE mRNAs involved in adipogenesis. DEmRNAs and DE circRNAs/lncRNAs are represented by circles and diamonds, respectively. The top 30 genes with the most connection were highlighted. The higher the connectivity, the larger the icon and the redder the color

To identify the key genes involved in IMF deposition, 61 DEMs were identified from fat deposition-related pathways in this study (Additional file 7). We then constructed a circRNA/lncRNA**–**mRNA coexpression network based on the screened DECs and DELs, and visualized the top 100 genes with the highest degree and their linked genes (Fig. 5 and Additional file 8). A total of 10 potential DECs and 108 key DELs that may be closely related to fat deposition were predicted and screened (Fig. 5). The findings revealed that the coexpression network included 891 connections and that each lncRNA might be associated with numerous mRNAs. A total of 2 circRNAs and 34 lncRNAs were discovered to be co-expressed with *FABP4*, 3 circRNAs and 42 lncRNAs were discovered to be coexpressed with *PLIN2*, and the network also comprised circRNAs/lncRNAs coexpressed with *DGAT2*, *C/EBPA*, *FOXO1*, *KLF4*, *FGF1* and *IL6*. These findings suggested that these circRNAs/lncRNAs may play significant roles in regulating adipogenesis.

### A miRNA landscape during intramuscular adipogenesis

**Fig. 6:**
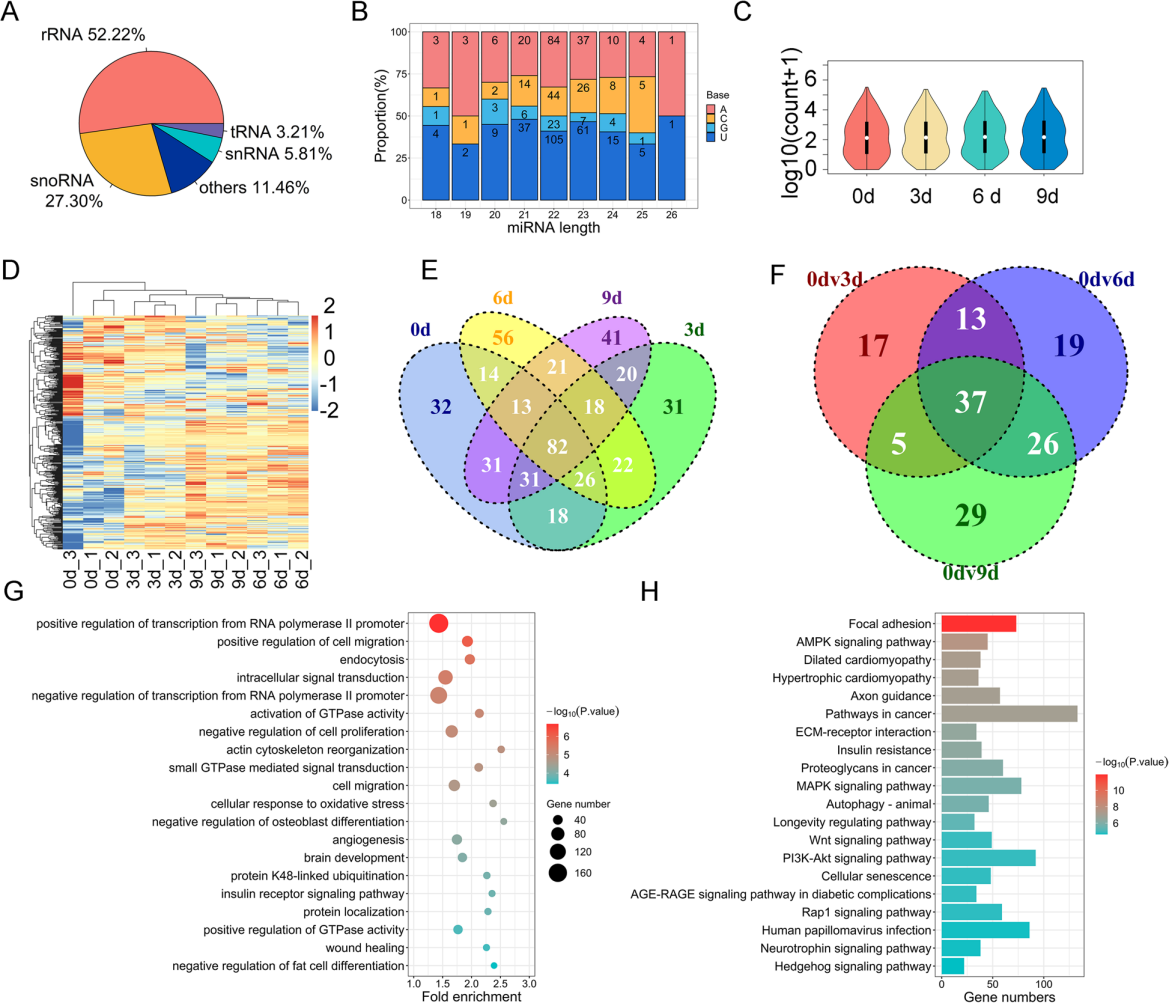
The landscape of miRNA. **A** The type and proportion of ncRNAs. **B** Initial base preference of different length miRNAs. **C** Violin plot of miRNAs expression levels. **D** Clustered expression heatmap of all miRNAs. **E** Venn diagram showing the specifically expressed and commonly expressed miRNAs in four adipogenic differentiation stages (0d, 3d, 6d and 9d). **F** Expression analysis of common DE miRNAs during the intramuscular preadipocyte differentiation. **G**, **H**) GO (**G**) and KEGG (**H**) enricment analysis of DE miRNAs

In the present study, we continued to perform miRNA-seq on samples from intramuscular preadipocyte differentiation Days 0, 3, 6, and 9. A total of 427 miRNAs were acquired from these four stages (0, 3, 6, and 9 d) of differentiation by removing other noncoding RNAs, such as rRNA and tRNA (Fig. 6A). The initial base of these miRNAs was uridine and they were mostly expressed at the middle (10 < count < 100) levels (Fig. 6B). As shown in Fig. 6C and D, we also drew a violin plot of miRNA expression and a heatmap of clustered expression. We then identified 32, 31, 56 and 41 specifically expressed miRNAs at different stages of differentiation, and there were 82 common miRNAs (Fig. 6E and Additional file 9). Differential expression analysis of 3dv0d, 6dv0d and 9dv0d revealed a total of 77 differentially expressed miRNAs (DEmiRs) during the adipogenic differentiation of intramuscular preadipocytes (Fig. 6F and Additional file 10). To further explore the functions of these DEmiRs, we performed functional enrichment analysis on the target genes of DEmiRs. The GO terms and KEGG signaling pathways of DEmiRs were mainly enriched in negative regulation of cell proliferation, insulin receptor signaling pathway, negative regulation of fat cell differentiation, AMPK signaling pathway, Insulin resistance, MAPK signaling pathway and PI3K-Akt signaling pathway (Fig. 6G, H and Additional file 11).

### Construction of the circRNA/lncRNA–miRNA–mRNA ceRNA network

**Fig. 7:**
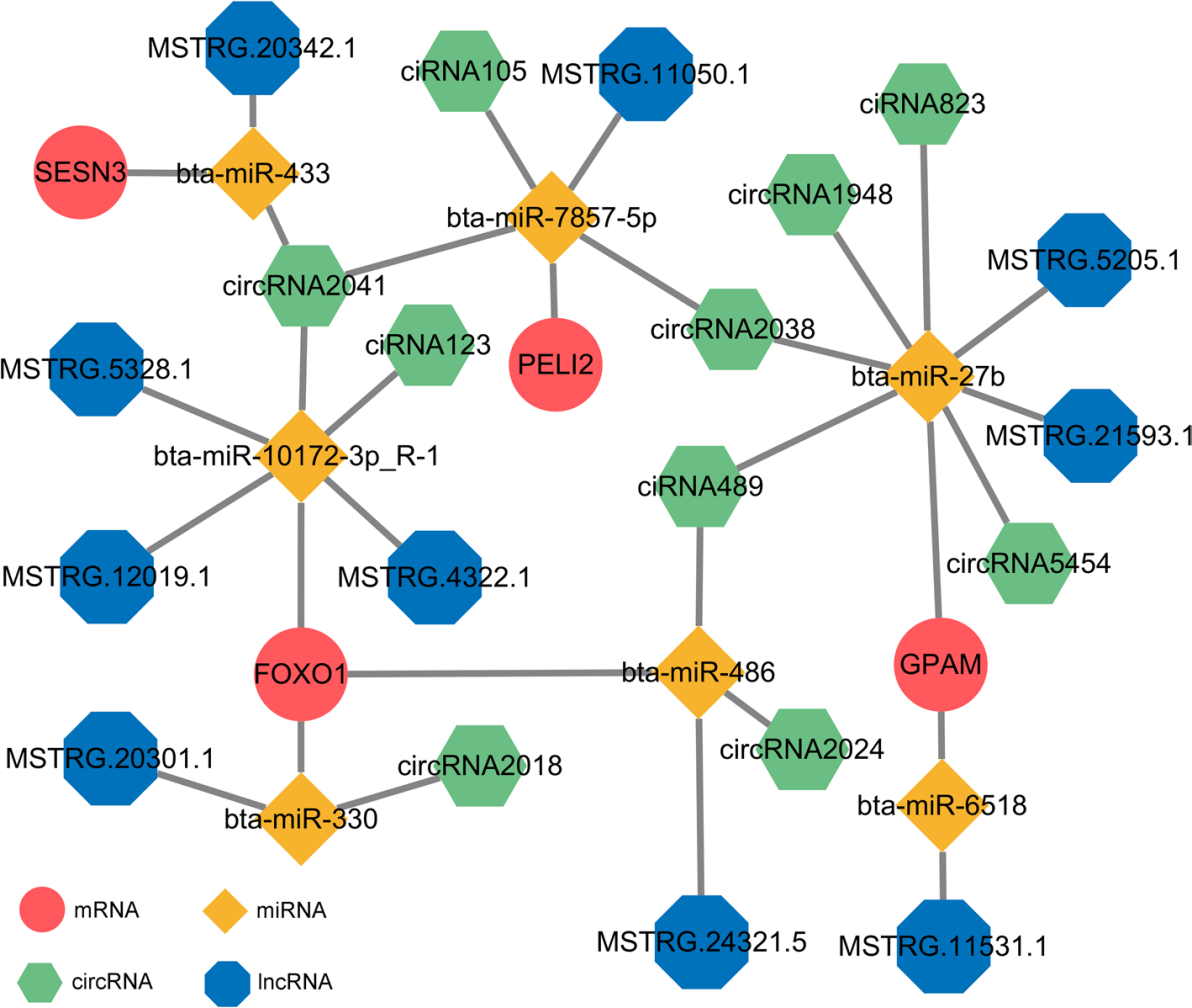
mRNA-miRNA-circRNA/lncRNA co-expression network. Co-expressed network of DE miRNAs and their targeted DE circRNAs/lncRNAs and DE mRNAs. DE mRNAs, DE circRNAs, DE lncRNAs and DE miRNAs are represented by circles (red), octagons (green), hexagons (blue) and rhombuses (yellow), respectively

The target gene prediction results of miRNAs were integrated into the previously established circRNA/lncRNA**–**mRNA coexpression network to further discover the key genes regulating IMF deposition. We analysed the DEmiRs involved in the ceRNA regulatory network and finally constructed a circRNA/lncRNA**–**miRNA**–**mRNA network based on the results of the circRNA/lncRNA**–**mRNA coexpression network (Fig. 7 and Additional file 12). The network contains 24 ceRNA axes, consisting of 4 mRNAs, 10 circRNAs, 10 lncRNAs and 7 miRNAs. Among them, *SESN3*, *FOXO1*, *GPAM*, and *miR-27b* were all related to lipid storage, activation of MAPK activity and fat cell differentiation [23–26], suggesting that the screened circRNAs/lncRNAs/miRNAs may play important roles in regulating adipogenesis (Fig. 7).

### Validation of differentially expressed genes, circRNAs, lncRNAs and miRNAs

**Fig. 8:**
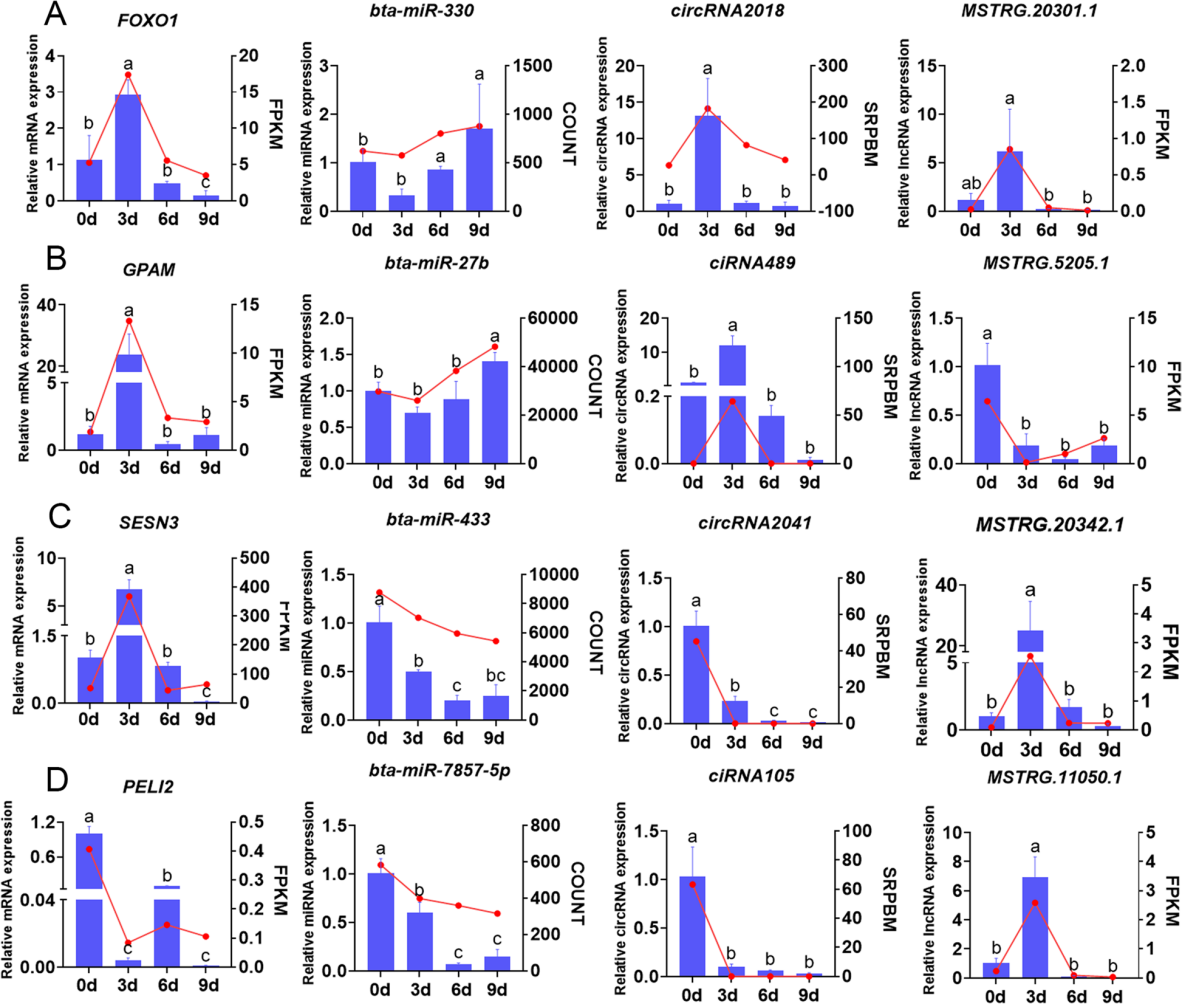
The expression level of four transcript types (mRNA, circRNA, lncRNA and miRNA) in the ceRNA network during bovine intramuscular preadipocyte differentiation. Data from qRT-PCR are shown as column (blue) and *Y*-axis on the left, while the data from RNA-seq are shown as line (red) and *Y*-axis on the right. n = 3. Different lowercase letters indicate *P* < 0.05 and the same letters indicate *P* > 0.05

All 31 genes in the ceRNA regulatory network were differentially expressed during intramuscular adipogenic differentiation (Fig. 7). Among them, 4 mRNAs (*SESN3*, *FOXO1*, *GPAM*, and *PELI2*), 4 circRNAs (*circRNA2018*, *ciRNA489*, *circRNA2627* and *ciRNA105*), 4 lncRNAs (*MSTRG.20301.1*, *MSTRG.5025.1*, *MSTRG.20342.1*, and *MSTRG.11050.1*) and 4 miRNAs (*bta-miR-330*, *bta-miR-27b*, *bta-miR-433*, *bta-miR-7857-5p*) were selected for qRT**–**PCR validation (Fig. 8). The expression trends of these genes during adipogenic differentiation were highly consistent with the results of RNA-seq, demonstrating the reliability of the RNA-seq data (Fig. 8).

## Discussion

A large body of evidence suggests that adipogenic differentiation of preadipocytes is precisely regulated by multiple cytokines, including transcription factors, signaling pathways and noncoding RNAs [27]. Some of these cytokines are well conserved functionally across species, but there is a subset of regulatory elements, such as lncRNAs, whose functions are not conserved across species [28]. This means that many previous studies focusing on humans and mice have lost their original reference value in the field of livestock production. Additionally, most studies on animal adipogenesis have focused on subcutaneous and perirenal fat [13, 15–21], with fewer studies on intramuscular adipose tissue. The origin and development of IMF are different from that of other adipose tissues, which makes it impossible to fully replicate the studies of other adipose tissues in IMF tissue as well [6–8]. Meanwhile, the molecular mechanisms of these regulatory cytokines have mostly been studied individually. However, they may in fact act synergistically in fat deposition in a more complex way. For example, a new study found that *circFLT1* and *lncCCPG1* could promote the expression of *lncSLC30A9* by adsorbing *miR-93*, and ultimately regulate the adipogenesis of bovine adipocytes through the AKT/FOS axis [29]. The above results indicate that the combined analysis of multiple regulatory elements is more beneficial for revealing the molecular mechanism and regulatory network of IMF deposition in beef cattle. In this study, whole transcriptome sequencing of the same batch of intramuscular adipocytes at different differentiation time points was performed to screen the key regulators involved in regulating bovine intramuscular preadipocyte differentiation, including mRNAs, circRNAs, lncRNAs and miRNAs.

In the present study, we annotated a total of 27,153 mRNAs, 14,070 circRNAs, 7035 lncRNAs and 427 miRNAs that were expressed during bovine intramuscular preadipocyte differentiation. The number of circRNAs annotated in this study was much higher than annotated in similar studies in bovine muscle tissue (1318 ciRNAs and 5177 circRNAs) [30, 31] and higher than the sequencing results of porcine subcutaneous adipocyte differentiation (8623 circRNAs) [21]. However, it is close to the recent circRNA-seq results of sheep muscle (11,749 circRNAs) [32]. In terms of the observed distribution of ncRNA types, the numbers of intergenic lncRNAs and canonical circRNAs accounted for the largest proportions of lncRNAs and circRNAs, respectively, and were similar to the results during porcine subcutaneous fat differentiation [21] and in line with the general rule [33, 34].

Studies have shown that the PI3K-Akt, MAPK, and FoxO signaling pathways regulate subcutaneous and visceral adipogenesis [35–38]. Recent studies have also found that these pathways are important for IMF deposition in pigs and cattle [39–41]. In this study, differentially expressed genes, including mRNAs and ncRNAs, were significantly enriched in these pathways, indicating their potential regulatory effects on bovine IMF deposition. We will explore the molecular mechanisms of these genes in the future. Meanwhile, we found that DEMs, DECs and DELs were significantly enriched in the Focal adhesion and the ECM-receptor-interaction pathways, which was consistent with the findings in goat and chicken IMF [42, 43]. Studies have found that focal adhesion kinase (FAK) and ECM-receptor interactions can regulate intramuscular adipogenesis in goats and chickens [43, 44]. IMF is rich in unsaturated fatty acids [4], and our results showed that DELs were significantly enriched in the α-linolenic acid and linoleic acid metabolism pathways. The results suggest that DELs may also be involved in fatty acid metabolism in bovine IMF deposition.

To identify key candidate genes regulating intramuscular adipocyte differentiation, we performed WCGNA on the annotated mRNAs. The differentially expressed mRNAs among them related to adipogenesis were further identified. The obtained DEMs were then combined with DELs and DECs for coexpression network analysis to construct a circRNA/lncRNA–mRNA regulatory network in intramuscular adipocyte differentiation. We found some genes in the network, such as *FABP4*, *PLIN2*, *DGAT2*, *FOXO1* and *CEPBA*, whose roles in adipogenesis are well known. Many studies have also found that ncRNAs interact with these genes to regulate adipogenesis. For example, lncRNA MIR31HG affected adipocyte differentiation by regulating histone modifications of the *FABP4* promoter [45]. The *lncRNA ADINR* has been reported to activate *CEBPA* expression at the transcriptional level to positively regulate adipogenic differentiation [46]. circEVI5 can bind miR-4793-3p as a ceRNA to promote *FOXO1* expression [47]. More interestingly, *CEBPA* can mediate the expression of *lncRNA PLIN2* from *PLIN2* transcripts to activate the Wnt pathway [48]. Additionally, we also identified the *SH2B2* gene in the coexpression network, and our previous studies have shown that the polymorphism of the *SH2B2* gene is closely related to the growth traits of cattle [49]. These studies suggest that the mRNAs and ncRNAs we screened may serve as candidate targets for follow-up studies.

miRNAs can directly target mRNAs to regulate gene expression and often interact with mRNAs and ncRNAs through the ceRNA network to play regulatory roles [50]. Through differential expression and functional enrichment analyses, we identified many miRNAs, including miRNA-30c [51], miR-25-3p [52] and miR-23a ~ 27a ~ 24-2 [53], that have been found to target key genes to regulate human and bovine adipogenesis. More importantly, our results revealed several potential ceRNA regulatory networks during bovine intramuscular preadipocyte differentiation. Among them, multiple studies have reported that *SESN3*, *FOXO1*, *GPAM*, and *miR-27b* regulate adipocyte differentiation and lipid metabolism [23–26]. Furthermore, the expression levels of *SESN3, PELI2, GPAM* and *FOXO1* have been found to be regulated by the competitive binding of lncRNAs/circRNAs and miRNAs in adipogenesis or other biological processes [23, 54–58]. Finally, we further verified the expression of the genes in the ceRNA network by qRT–PCR, which demonstrated the reliability of the sequencing results in this study. Apparently, the expression trends of mRNAs and circRNAs in the ceRNA networks were largely consistent, whereas the expression patterns of miRNAs were opposite (e.g., *FOXO1*/bta-miR-330/circRNA2018, *GPAM*/bta-miR-27b/circRNA489 and *SESN3*/bta-miR-433/circRNA2627), implying that these miRNAs may target and repress the expression of these mRNAs and circRNAs. These results suggest that the genes we screened have important roles in regulating bovine IMF deposition. We will also continue to verify the specific mechanism of action of these candidate mRNAs and noncoding RNAs.

## Conclusion

In conclusion, genome-wide identification of mRNAs, lncRNAs, circRNAs and miRNAs during bovine intramuscular adipogenesis was performed using whole transcriptome sequencing. Finally, 31 key potential genes, including 4 mRNAs, 10 circRNAs, 10 lncRNAs and 7 miRNAs, related to adipogenesis were screened and identified through WGCNA, coexpression network analysis and ceRNA mechanism analysis. Our results provide new candidate molecular markers for the selection and improvement of beef cattle, and provide a theoretical basis for studying the molecular mechanisms of key noncoding RNAs in intramuscular preadipocyte differentiation.

## Material and methods

### Isolation, culture and differentiation of bovine intramuscular preadipocytes

Intramuscular preadipocytes from the longissimus dorsi muscle in newborn Qinchuan cattle were isolated using the methods of separating intramuscular preadipocytes established in our laboratory [59]. Briefly, minced longissimus dorsi muscles were digested with collagenase type I (Gibco, NY, USA) for 1 h at 30 °C. Neutralize the digestion solution with an equal volume of DMEM/F12 medium (BI, MA, USA) containing 15% FBS (PAN-Seratech, Germany) and 1% penicillin/streptomycin (Hyclone, UT, USA) (i.e., growth medium). Pass through a cell sieve and centrifuge at 700 *g* for 10 min. Add serum-free DMEM/F12 to the sediment and wash 2 times and centrifuge at 700 *g* for 10 min. After resuspending the cells with DMEM/F12 medium containing 15% FBS, inoculate them in cell culture dishes. After incubation at 37 °C and 5% CO_2_ for 2 h, the medium and unadhered cells were removed. Wash with PBS 3 times and new growth medium was added and the medium was changed every 2 days. Adipogenic differentiation was performed by replacing complete medium containing 0.5 mM 3-Isobutyi-1-methylxanthine (IBMX, Sigma, MO, USA), 1 μM dexamethasone (Sigma) and 2 μM insulin (Sigma) when intramuscular preadipocytes reached 90% confluency, and 2 days later using complete medium containing 2 μM insulin to maintains adipogenic differentiation.

### Oil red O staining

After removal of the culture medium, the intramuscular preadipocytes were fixed with 4% paraformaldehyde (Solarbio, Beijing, China) and stained with Oil Red O dye solution (Solarbio) for 30 min. After washing off excess Oil Red O with PBS, photographs were taken using an Olympus IX71 microscope (Olympus Corporation, Tokyo, Japan).

### Cellular triglyceride assay

The levels of cellular triglyceride during the adipogenic differentiation of bovine intramuscular adipocytes were detected using the Tissue Triglyceride (TG) Content Assay Kit (Applygen Technologies, Beijing, China). Total protein concentrations were determined by the TaKaRa BCA Protein Assay Kit (Takara). All of the experiments were performed according to the manufacturer’s recommended protocol. The values obtained were normalized to the total cellular protein content and were expressed as nmol/μg protein.

### RNA isolation, library preparation, and sequencing

Intramuscular adipocytes on day 0, 3, 6 and 9 of differentiation were harvested. Cells at each time point included 3 biological replicates. RNAiso Plus Kit (Takara, Beijing, China) was used to extract cellular total RNA. NanoQuantplate™ (Infinite M200 PRO, TECAN, Switzerland) was used to measure the concentration and purity of RNA. The certified RNA was stored at 80 °C for subsequent use. Then, the RNA samples were sent to LC-BIO Bio-tech ltd (Hangzhou, China) for RNA sequencing. Strand-specific libraries were created via rRNA depletion. The average insert size for the final cDNA library was 300 bp (±50 bp). At last, we performed the paired-end 2 × 150 bp sequencing on an Illumina Novaseq™ 6000 platform at the LC-BIO Bio-tech ltd (Hangzhou, China) following the vendor’s recommended protocol.

### Identification of circRNA, lncRNA and miRNA

Trimmomatic [60] was used to remove adaptor contamination, low-quality bases, and uncertain bases from sequencing reads. FastQC v0.11.9 was used to check the sequence quality. Bowtie2 2.2.3 [61] and Hisat2 2.1.0 [62] were used to map the reads to the genome of the *Bos taurus* (ARS-UCD1.2).

Identification of circRNA: CIRCExplorer2 [63, 64] and CIRI [65] were used to denovo assemble the mapped reads to circular RNAs first. Then, tophat-fusion was used to identify back splicing reads in unmapped reads. Unique circular RNAs were produced for each sample. The above software uses default parameters for calculation.

Identification of lncRNA: After removed the other non-coding RNAs (ribosomal RNA (rRNA), transfer RNA (tRNA), small nucleolar RNA (snoRNA), and small nuclear RNA (snRNA)), the transcripts that overlapped with known mRNAs were eliminated, as were transcripts shorter than 200 bp. Then, to identify transcripts with coding potential. All transcripts with a CPC < − 1 and a CNCI < 0 were discarded [66, 67]. The remaining transcripts were classified as long non-coding RNAs (lncRNAs).

Identification of miRNA: ACGT101-miR (LC Sciences, Houston, Texas, USA) was used to eliminate adaptor dimers, trash, low complexity, common RNA families (rRNA, tRNA, snRNA, snoRNA), and repetitions from raw readings. Subsequently, BLAST searches were used to map unique sequences with lengths of 18-26 nucleotides to specific species precursors in miRBase 22.0 [68] in order to find recognized miRNAs and novel 3p- and 5p-derived miRNAs. The alignment allowed for length variance at both the 3′ and 5′ ends, and one mismatch inside the sequence. The hairpin arms with unique sequences matching to specific species mature miRNAs were recognized as known miRNAs. The sequences that mapped to the other arm of a known specific species precursor hairpin opposite the annotated mature miRNA-containing arm were novel 5p- or 3p-derived miRNA candidates.

### Analysis of differentially expressed genes (DEGs)

The fragments per kilobase of transcript per million reads mapped (FPKM) value was used to estimate the expression levels of mRNAs and lncRNAs, while the spliced reads per billion mapping (SRPBM) value was utilized to determine the amount of circRNAs, and the normalized raw counts was utilized to measure the number of miRNAs. The R package-edgeR provides statistical procedures for determining differential expression in digital gene expression data using models based on the negative binomial distribution. The *p*-values are adjusted using quasi-likelihood method to control the false discovery rates. Normalized data using Weighted trimmed mean of M-values (TMM). In three comparisons (3d versus 0d, 6d versus 0d, and 9d versus 0d), the differentially expressed genes, including mRNAs, circRNAs, lncRNAs and miRNAs, were selected with log2 (fold change) > 1 or log2 (fold change) < − 1 and adj *P* value < 0.05 by R package–edgeR [69].

### Screening potential target gene of differentially expressed circRNA/lncRNA/miRNA

According to the diverse function modes of circRNA, lncRNA, and miRNA, three screening methodologies were devised. For differentially expressed circRNAs (DECs), the host gene of them was regarded as their potential target genes. For differentially expressed lncRNAs (DELs), screened based on their genomic positional relation 100 kilobase pairs (kb) upstream and 100 kb downstream transcripts as cis-target mRNAs of lncRNAs. For DE miRNAs, obtained potential target genes of differentially expressed miRNAs (DEmiRs) based on TargetScan 8.0 [70] and miRanda 3.3 [71], and the mRNA/circRNA/lncRNA-miRNA pairs which TargetScan score > 90 and miranda energy < − 20 were reserved.

### GO and KEGG functional enrichment analysis

GO biological function enrichment analysis and KEGG signaling pathway enrichment analysis of genes were analyzed using DAVID 2021 online tool [72, 73] to explain the role of differentially expressed mRNAs (DEMs) and potential target genes of DECs, DELs and DEmiRs in the adipogenic differentiation of bovine intramuscular preadipocytes. The *p*-value of GO and KEGG enrichment was adjusted by the Fisher’s Exact Test method. The R program ggplot2 3.3.5 exhibited the top 20 adj *P*-value GO terms and signaling pathways.

### Weighted gene co-expression network analysis (WGCNA)

The weighted gene co-expression network was established using WGCNA 1.69, an R program [74]. The variance degree of each gene expression level among samples was calculated using the standardized gene expression matrix as input. The expression matrix was standardized by log2(FPKM+ 1), the top 75% of genes with the median absolute deviation (MAD) were chosen, and genes with expression levels MAD of less than 0.01 were removed in each sample. After threshold screening, power processing with a = 4 returned the scaleless adjacency matrix. The adjacency matrix was transformed into a topological overlap matrix (ToM), and the dynamic cutting technique was used to cluster and split genes using the topological difference matrix (distom = 1-tom) in order to further examine the correlation of gene expression patterns. The modules with more than 75% similarity were merged by using the default tree height cut of 0.25: MEDISSTHRES = 0.25 in WGCNA.

### Co-expression network construction

Based on possible cis-acting, host relationship, the Pearson correlation coefficient of circRNA/lncRNA-mRNA, prediction of miRNA target genes and protein-protein interaction analysis, Cytoscape software 3.8.0 [75] was used to construct the co-expression regulatory network, including DEMs, DECs and DELs, with potentially important roles in adipogenic differentiation. DECs and DELs were chosen based on host-regulatory relationships, cis-regulatory relationships, and gene expression level Pearson correlation coefficients > 0.95. The MCC plug-in built in cytoHubba was used to mark the top 30 connected nodes according to default parameters.

### Quantitative real-time PCR (qRT-PCR)

Three RNA samples per differentiation stage were reverse transcribed according to the manufacturer’s instructions using PrimeScrip RT reagent Kit with gDNA Eraser (Perfect Real Time) (Takara) and miRcute Plus miRNA First-Strand cDNA Kit (TIANGEN, Beijing, China), respectively. The CFX Connect Real-Time PCR Detection System was used to perform qRT-PCR using TB Green Premix Ex Taq II (Tli RNaseH Plus) (Takara) and miRNA Plus miRNA qPCR Kit (TIANGEN), respectively. Additional file 13 contains a list of the primers used in qRT-PCR. Meanwhile, *β–actin* and *U6* was used as the normalization controls, respectively. The 2^-ΔΔCT^ method [76] was used to calculate the relative expression levels.

### Statistical analysis

All samples were prepared with 3 biological replicates, each including 3 technical replicates. Data are expressed as mean ± SD. GraphPad Prism 9.00 (CA, USA) was used for statistical analysis and graphing. *P* values were calculated using one-way ANOVA with Tukey’s correction among multiple groups, where *P < 0.05 and **P < 0.01 indicate significant differences.

## Supplementary Information

**Additional file 1: Supplementary Table S1.** Summary of sequencing data and read-alignment statistics from RNA-seq.

**Additional file 2: Supplementary Table S2.** List of mRNAs, circRNAs and lncRNAs during bovine intramuscular preadipocyte differentiation.

**Additional file 3: Supplementary Table S3.** DE mRNAs during intramuscular preadipocyte differentiation.

**Additional file 4: Supplementary Table S4.** DE circRNAs during intramuscular preadipocyte differentiation.

**Additional file 5: Supplementary Table S5.** DE lncRNAs during intramuscular preadipocyte differentiation.

**Additional file 6: Supplementary Table S6.** GO&KEGG analysis of DE mRNAs circRNAs lncRNAs.

**Additional file 7: Supplementary Table S7.** WGCNA result of DEMs.

**Additional file 8: Supplementary Table S8.** Co-expression network of DEMs, DECs and DELs involved in adipogenesis.

**Additional file 9: Supplementary Table S9.** List of miRNAs during bovine intramuscular preadipocyte differentiation.

**Additional file 10: Supplementary Table S10.** DE miRNAs during bovine intramuscular preadipocyte differentiation.

**Additional file 11: Supplementary Table S11.** GO&KEGG analysis of DE miRNAs.

**Additional file 12: Supplementary Table S12.** ceRNA networks of DEMs, DECs, DELs and DEmiRs involved in adipogenesis.

**Additional file 13: Supplementary Table S13.** Primers used in this study.

## Data Availability

The RNA-seq data generated during the current study are available in NCBI GEO repository with accession number GSE185850. All data analyzed during this study are included in this published article and the Additional files.

## Abbreviations

ncRNA: Non-coding RNA
circRNA: Circular RNA
lncRNA: Long non-coding RNAs
miRNA: Micro RNA
ciRNA: Circular intronic RNA
ceRNA: Competing endogenous RNAs
IMF: Intramuscular fat
PPARG: Peroxisome proliferator activated receptor gamma
C/EBPA: CCAAT enhancer binding protein alpha
FABP4: Fatty acid binding protein 4
CDC10: Septin 7
WGCNA: Weighted gene co-expression network analysis
FBS: Fetal bovine serum
CIRI: CircRNA identifier
CNCI: Coding-non-coding index
CPC: Coding potential calculator
GO: Gene ontology
KEGG: Kyoto Encyclopedia of Genes and Genomes
SnRNA: Small nuclear RNA
SnoRNA: Small nucleolar RNA
ANOVA: Analysis of variance

## References

[1] Stewart SM, Lauridsen T, Toft H, Pethick DW, Gardner GE, McGilchrist P (2021). Objective grading of eye muscle area, intramuscular fat and marbling in Australian beef and lamb. Meat Sci.

[2] Stewart SM, Gardner GE, McGilchrist P, Pethick DW, Polkinghorne R, Thompson JM (2021). Prediction of consumer palatability in beef using visual marbling scores and chemical intramuscular fat percentage. Meat Sci.

[3] Fortin A, Robertson WM, Tong AK (2005). The eating quality of Canadian pork and its relationship with intramuscular fat. Meat Sci.

[4] Joo ST, Hwang YH, Frank D (2017). Characteristics of Hanwoo cattle and health implications of consuming highly marbled Hanwoo beef. Meat Sci.

[5] Baik M, Kang HJ, Park SJ, Na SW, Piao M, Kim SY (2017). Triennial growth and development symposium: molecular mechanisms related to bovine intramuscular fat deposition in the longissimus muscle. J Anim Sci.

[6] Taga H, Bonnet M, Picard B, Zingaretti MC, Cassar-Malek I, Cinti S (2011). Adipocyte metabolism and cellularity are related to differences in adipose tissue maturity between Holstein and Charolais or blond d'Aquitaine fetuses. J Anim Sci.

[7] Keogh K, Kelly AK, Kenny DA (2021). Effect of plane of nutrition in early life on the transcriptome of visceral adipose tissue in Angus heifer calves. Sci Rep.

[8] Du M, Huang Y, Das AK, Yang Q, Duarte MS, Dodson MV (2013). Meat science and muscle biology symposium: manipulating mesenchymal progenitor cell differentiation to optimize performance and carcass value of beef cattle. J Anim Sci.

[9] Fu YY, Chen KL, Li HX, Zhou GH (2016). The adipokine Chemerin induces lipolysis and adipogenesis in bovine intramuscular adipocytes. Mol Cell Biochem.

[10] Tong B, Gao GQ, Muramatsu Y, Ohta T, Kose H, Li GP (2015). Association of the expression levels in the longissimus muscle and a SNP in the CDC10 gene with marbling in Japanese black beef cattle. Meat Sci.

[11] Adelman K, Egan E (2017). Non-coding RNA: more uses for genomic junk. Nature.

[12] Boivin V, Reulet G, Boisvert O, Couture S, Elela SA, Scott MS (2020). Reducing the structure bias of RNA-Seq reveals a large number of non-annotated non-coding RNA. Nucleic Acids Res.

[13] Sun L, Goff LA, Trapnell C, Alexander R, Lo KA, Hacisuleyman E (2013). Long noncoding RNAs regulate adipogenesis. Proc Natl Acad Sci U S A.

[14] Bartel DP (2004). MicroRNAs: genomics, biogenesis, mechanism, and function. Cell.

[15] Chen X, Raza SHA, Ma X, Wang J, Wang X, Liang C (2021). Bovine pre-adipocyte Adipogenesis is regulated by bta-miR-150 through mTOR signaling. Front Genet.

[16] Chen X, Raza SHA, Cheng G, Ma X, Wang J, Zan L. Bta-miR-376a targeting KLF15 interferes with Adipogenesis signaling pathway to promote differentiation of Qinchuan beef cattle Preadipocytes. Animals (Basel). 2020;10(12). 10.3390/ani10122362. 10.3390/ani10122362 PMC7763857 33321855

[17] Jiang R, Li H, Yang J, Shen X, Song C, Yang Z (2020). circRNA profiling reveals an abundant circFUT10 that promotes adipocyte proliferation and inhibits adipocyte differentiation via sponging let-7. Mol Ther Nucleic Acids.

[18] Shen X, Tang J, Ru W, Zhang X, Huang Y, Lei C (2020). CircINSR regulates fetal bovine muscle and fat development. Front Cell Dev Biol.

[19] Zhang S, Kang Z, Cai H, Jiang E, Pan C, Dang R (2021). Identification of novel alternative splicing of bovine lncRNA lncFAM200B and its effects on preadipocyte proliferation. J Cell Physiol.

[20] Cai H, Li M, Jian W, Song C, Huang Y, Lan X (2020). A novel lncRNA BADLNCR1 inhibits bovine adipogenesis by repressing GLRX5 expression. J Cell Mol Med.

[21] Liu X, Liu K, Shan B, Wei S, Li D, Han H (2018). A genome-wide landscape of mRNAs, lncRNAs, and circRNAs during subcutaneous adipogenesis in pigs. J Anim Sci Biotechnol.

[22] Wang J, Ren Q, Hua L, Chen J, Zhang J, Bai H, et al. Comprehensive analysis of differentially expressed mRNA, lncRNA and circRNA and their ceRNA networks in the Longissimus Dorsi muscle of two different pig breeds. Int J Mol Sci. 2019;20(5). 10.3390/ijms20051107. 10.3390/ijms20051107 PMC6429497 30836719

[23] Lin W, Zhao J, Yan M, Li X, Yang K, Wei W, et al. SESN3 inhibited SMAD3 to relieve its suppression for MiR-124, thus regulating pre-adipocyte Adipogenesis. Genes (Basel). 2021;12(12). 10.3390/genes12121852. 10.3390/genes12121852 PMC8701261 34946801

[24] Ioannilli L, Ciccarone F, Ciriolo MR. Adipose tissue and FoxO1: bridging physiology and mechanisms. Cells. 2020;9(4). 10.3390/cells9040849. 10.3390/cells9040849 PMC7226803 32244542

[25] Yu H, Zhao Z, Yu X, Li J, Lu C, Yang R (2017). Bovine lipid metabolism related gene GPAM: molecular characterization, function identification, and association analysis with fat deposition traits. Gene.

[26] Karbiener M, Fischer C, Nowitsch S, Opriessnig P, Papak C, Ailhaud G (2009). microRNA miR-27b impairs human adipocyte differentiation and targets PPARgamma. Biochem Biophys Res Commun.

[27] Ghaben AL, Scherer PE (2019). Adipogenesis and metabolic health. Nat Rev Mol Cell Biol.

[28] Rinn JL, Chang HY (2020). Long noncoding RNAs: molecular modalities to organismal functions. Annu Rev Biochem.

[29] Kang Z, Zhang S, Jiang E, Wang X, Wang Z, Chen H (2020). circFLT1 and lncCCPG1 sponges miR-93 to regulate the proliferation and differentiation of adipocytes by promoting lncSLC30A9 expression. Mol Ther Nucleic Acids.

[30] Chen M, Wei X, Song M, Jiang R, Huang K, Deng Y (2021). Circular RNA circMYBPC1 promotes skeletal muscle differentiation by targeting MyHC. Mol Ther Nucleic Acids.

[31] Yan XM, Zhang Z, Meng Y, Li HB, Gao L, Luo D (2020). Genome-wide identification and analysis of circular RNAs differentially expressed in the longissimus dorsi between Kazakh cattle and Xinjiang brown cattle. PeerJ.

[32] Bao G, Zhao F, Wang J, Liu X, Hu J, Shi B (2022). Characterization of the circRNA-miRNA-mRNA network to reveal the potential functional ceRNAs associated with dynamic changes in the meat quality of the Longissimus Thoracis muscle in Tibetan sheep at different growth stages. Front Vet Sci.

[33] La Y, He X, Zhang L, Di R, Wang X, Gan S, et al. Comprehensive analysis of differentially expressed profiles of mRNA, lncRNA, and circRNA in the uterus of seasonal reproduction sheep. Genes (Basel). 2020;11(3). 10.3390/genes11030301. 10.3390/genes11030301 PMC7140836 32178360

[34] Jin L, Tang Q, Hu S, Chen Z, Zhou X, Zeng B (2021). A pig BodyMap transcriptome reveals diverse tissue physiologies and evolutionary dynamics of transcription. Nat Commun.

[35] Cignarelli A, Genchi VA, Perrini S, Natalicchio A, Laviola L, Giorgino F. Insulin and insulin receptors in adipose tissue development. Int J Mol Sci. 2019;20(3). 10.3390/ijms20030759. 10.3390/ijms20030759 PMC6387287 30754657

[36] Xiao F, Tang CY, Tang HN, Wu HX, Hu N, Li L (2021). Long non-coding RNA 332443 inhibits Preadipocyte differentiation by targeting Runx1 and p38-MAPK and ERK1/2-MAPK signaling pathways. Front Cell Dev Biol.

[37] Yang JT, Chen YJ, Huang CW, Wang YC, Mersmann HJ, Wang PH, et al. Docosahexaenoic acid suppresses expression of Adipogenic Tetranectin through sterol regulatory element-binding protein and Forkhead box O protein in pigs. Nutrients. 2021;13(7). 10.3390/nu13072315. 10.3390/nu13072315 PMC8308646 34371822

[38] Zhao Z, Deng X, Jia J, Zhao L, Wang C, Cai Z (2022). Angiopoietin-like protein 8 (betatrophin) inhibits hepatic gluconeogenesis through PI3K/Akt signaling pathway in diabetic mice. Metabolism.

[39] Zhao C, Chen X, Wu W, Wang W, Pang W, Yang G (2016). MAT2B promotes adipogenesis by modulating SAMe levels and activating AKT/ERK pathway during porcine intramuscular preadipocyte differentiation. Exp Cell Res.

[40] Poleti MD, Regitano LCA, Souza G, Cesar ASM, Simas RC, Silva-Vignato B (2018). Longissimus dorsi muscle label-free quantitative proteomic reveals biological mechanisms associated with intramuscular fat deposition. J Proteome.

[41] Shi B, Shi X, Zuo Z, Zhao S, Zhao Z, Wang J (2022). Identification of differentially expressed genes at different post-natal development stages of longissimus dorsi muscle in Tianzhu white yak. Gene.

[42] Xiong Y, Wang Y, Xu Q, Li A, Yue Y, Ma Y (2021). LKB1 regulates goat intramuscular Adipogenesis through focal adhesion pathway. Front Physiol.

[43] Luo N, Shu J, Yuan X, Jin Y, Cui H, Zhao G (2022). Differential regulation of intramuscular fat and abdominal fat deposition in chickens. BMC Genomics.

[44] San J, Du Y, Wu G, Xu R, Yang J, Hu J (2021). Transcriptome analysis identifies signaling pathways related to meat quality in broiler chickens - the extracellular matrix (ECM) receptor interaction signaling pathway. Poult Sci.

[45] Huang Y, Jin C, Zheng Y, Li X, Zhang S, Zhang Y (2017). Knockdown of lncRNA MIR31HG inhibits adipocyte differentiation of human adipose-derived stem cells via histone modification of FABP4. Sci Rep.

[46] Xiao T, Liu L, Li H, Sun Y, Luo H, Li T (2015). Long noncoding RNA ADINR regulates Adipogenesis by transcriptionally activating C/EBPalpha. Stem Cell Reports.

[47] Yan M, Niu L, Liu J, Yao Y, Li H (2021). circEVI5 acts as a miR-4793-3p sponge to suppress the proliferation of gastric cancer. Cell Death Dis.

[48] Sun C, Luan S, Zhang G, Wang N, Shao H, Luan C (2017). CEBPA-mediated upregulation of the lncRNA PLIN2 promotes the development of chronic myelogenous leukemia via the GSK3 and Wnt/beta-catenin signaling pathways. Am J Cancer Res.

[49] Raza SHA, Khan R, Gui L, Schreurs NM, Wang X, Mei C (2020). Bioinformatics analysis and genetic polymorphisms in genomic region of the bovine SH2B2 gene and their associations with molecular breeding for body size traits in qinchuan beef cattle. Biosci Rep.

[50] Yamamura S, Imai-Sumida M, Tanaka Y, Dahiya R (2018). Interaction and cross-talk between non-coding RNAs. Cell Mol Life Sci.

[51] Karbiener M, Neuhold C, Opriessnig P, Prokesch A, Bogner-Strauss JG, Scheideler M (2011). MicroRNA-30c promotes human adipocyte differentiation and co-represses PAI-1 and ALK2. RNA Biol.

[52] Zhang F, Xiong Q, Tao H, Liu Y, Zhang N, Li XF (2021). ACOX1, regulated by C/EBPalpha and miR-25-3p, promotes bovine preadipocyte adipogenesis. J Mol Endocrinol.

[53] Wang Y, Zhang Y, Su X, Wang H, Yang W, Zan L. Cooperative and independent functions of the miR-23a~27a~24-2 cluster in bovine adipocyte Adipogenesis. Int J Mol Sci. 2018;19(12). 10.3390/ijms19123957. 10.3390/ijms19123957 PMC6321175 30544847

[54] Li S, Yang S, Qiu C, Sun D (2021). LncRNA MSC-AS1 facilitates lung adenocarcinoma through sponging miR-33b-5p to up-regulate GPAM. Biochem Cell Biol.

[55] Yang L, Yang F, Zhao H, Wang M, Zhang Y (2019). Circular RNA circCHFR facilitates the proliferation and migration of vascular smooth muscle via miR-370/FOXO1/Cyclin D1 pathway. Mol Ther Nucleic Acids.

[56] Li F, Li D, Zhang M, Sun J, Li W, Jiang R (2019). miRNA-223 targets the GPAM gene and regulates the differentiation of intramuscular adipocytes. Gene.

[57] You Q, Wang J, Jia D, Jiang L, Chang Y, Li W (2020). MiR-802 alleviates lipopolysaccharide-induced acute lung injury by targeting Peli2. Inflamm Res.

[58] Lin W, Tang Y, Zhao Y, Zhao J, Zhang L, Wei W (2020). MiR-144-3p targets FoxO1 to reduce its regulation of Adiponectin and promote Adipogenesis. Front Genet.

[59] Yang W, Tang K, Wang Y, Zhang Y, Zan L (2017). Melatonin promotes triacylglycerol accumulation via MT2 receptor during differentiation in bovine intramuscular preadipocytes. Sci Rep.

[60] Bolger AM, Lohse M, Usadel B (2014). Trimmomatic: a flexible trimmer for Illumina sequence data. Bioinformatics.

[61] Langmead B, Trapnell C, Pop M, Salzberg SL (2009). Ultrafast and memory-efficient alignment of short DNA sequences to the human genome. Genome Biol.

[62] Kim D, Langmead B, Salzberg SL (2015). HISAT: a fast spliced aligner with low memory requirements. Nat Methods.

[63] Zhang XO, Dong R, Zhang Y, Zhang JL, Luo Z, Zhang J (2016). Diverse alternative back-splicing and alternative splicing landscape of circular RNAs. Genome Res.

[64] Zhang XO, Wang HB, Zhang Y, Lu X, Chen LL, Yang L (2014). Complementary sequence-mediated exon circularization. Cell.

[65] Gao Y, Wang J, Zhao F (2015). CIRI: an efficient and unbiased algorithm for de novo circular RNA identification. Genome Biol.

[66] Kong L, Zhang Y, Ye ZQ, Liu XQ, Zhao SQ, Wei L (2007). CPC: assess the protein-coding potential of transcripts using sequence features and support vector machine. Nucleic Acids Res.

[67] Sun L, Luo H, Bu D, Zhao G, Yu K, Zhang C (2013). Utilizing sequence intrinsic composition to classify protein-coding and long non-coding transcripts. Nucleic Acids Res.

[68] Kozomara A, Birgaoanu M, Griffiths-Jones S (2019). miRBase: from microRNA sequences to function. Nucleic Acids Res.

[69] Robinson MD, McCarthy DJ, Smyth GK (2010). edgeR: a bioconductor package for differential expression analysis of digital gene expression data. Bioinformatics.

[70] McGeary SE, Lin KS, Shi CY, Pham TM, Bisaria N, Kelley GM, et al. The biochemical basis of microRNA targeting efficacy. Science. 2019;366(6472). 10.1126/science.aav1741. 10.1126/science.aav1741 PMC7051167 31806698

[71] Betel D, Koppal A, Agius P, Sander C, Leslie C (2010). Comprehensive modeling of microRNA targets predicts functional non-conserved and non-canonical sites. Genome Biol.

[72] Sherman BT, Hao M, Qiu J, Jiao X, Baseler MW, Lane HC, et al. DAVID: a web server for functional enrichment analysis and functional annotation of gene lists (2021 update). Nucleic Acids Res. 2022. 10.1093/nar/gkac194. 10.1093/nar/gkac194 PMC9252805 35325185

[73] Kanehisa M, Furumichi M, Sato Y, Ishiguro-Watanabe M, Tanabe M (2021). KEGG: integrating viruses and cellular organisms. Nucleic Acids Res.

[74] Langfelder P, Horvath S (2008). WGCNA: an R package for weighted correlation network analysis. BMC Bioinformatics.

[75] Shannon P, Markiel A, Ozier O, Baliga NS, Wang JT, Ramage D (2003). Cytoscape: a software environment for integrated models of biomolecular interaction networks. Genome Res.

[76] Livak KJ, Schmittgen TD (2001). Analysis of relative gene expression data using real-time quantitative PCR and the 2(−Delta Delta C(T)) method. Methods.

[77] Percie du Sert N, Hurst V, Ahluwalia A, Alam S, Avey MT, Baker M (2020). The ARRIVE guidelines 2.0: updated guidelines for reporting animal research. PLoS Biol.

